# Kokosanolide from the seed of *Lansium domesticum *Corr.

**DOI:** 10.1107/S1600536809008009

**Published:** 2009-03-14

**Authors:** Tri Mayanti, Unang Supratman, Mat Ropi Mukhtar, Khalijah Awang, Seik Weng Ng

**Affiliations:** a Department of Chemistry, Faculty of Mathematics and Natural Sciences, Padjadjaran University, Jatinangor 45363, Indonesia; b Department of Chemistry, University of Malaya, 50603 Kuala Lumpur, Malaysia

## Abstract

In the title compound, [systematic name: 8,14-secogammacera-7,14(27)-diene-3,21-dione], C_27_H_32_O_9_, each of the six-membered rings adopts the common chair conformation. In the crystal, mol­ecules are linked by an O–H⋯O_ester_ hydrogen bond into a helical chain.

## Related literature

For compounds from this plant species, see: Habaguchi *et al.* (1968 ▶); Kiang *et al.* (1967 ▶); Nishizawa *et al.* (1982 ▶, 1983 ▶, 1984 ▶, 1985 ▶, 1988 ▶); Saewan *et al.* (2006 ▶).
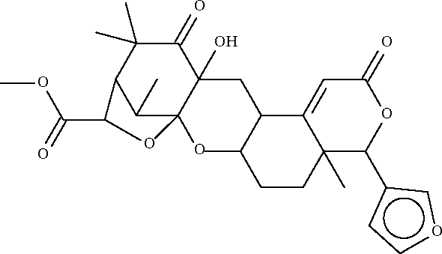


## Experimental

### 

#### Crystal data

C_27_H_32_O_9_
*M* *_r_* = 500.53
Monoclinic, 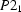

*a* = 8.8326 (1) Å
*b* = 12.8977 (2) Å
*c* = 11.1555 (1) Å
β = 110.777 (1)°
*V* = 1188.19 (3) Å^3^
*Z* = 2
Mo *K*α radiation
μ = 0.11 mm^−1^
*T* = 123 K
0.40 × 0.30 × 0.10 mm

#### Data collection

Bruker SMART APEX diffractometer
Absorption correction: none
11333 measured reflections
2856 independent reflections
2760 reflections with *I* > 2σ(*I*)
*R*_int_ = 0.020

#### Refinement

*R*[*F* ^2^ > 2σ(*F* ^2^)] = 0.030
*wR*(*F* ^2^) = 0.083
*S* = 1.03
2856 reflections
331 parameters
1 restraint
H-atom parameters constrained
Δρ_max_ = 0.32 e Å^−3^
Δρ_min_ = −0.21 e Å^−3^

### 

Data collection: *APEX2* (Bruker, 2007 ▶); cell refinement: *SAINT* (Bruker, 2007 ▶); data reduction: *SAINT*; program(s) used to solve structure: *SHELXS97* (Sheldrick, 2008 ▶); program(s) used to refine structure: *SHELXL97* (Sheldrick, 2008 ▶); molecular graphics: *X-SEED* (Barbour, 2001 ▶); software used to prepare material for publication: *publCIF* (Westrip, 2009 ▶).

## Supplementary Material

Crystal structure: contains datablocks global, I. DOI: 10.1107/S1600536809008009/bt2893sup1.cif

Structure factors: contains datablocks I. DOI: 10.1107/S1600536809008009/bt2893Isup2.hkl

Additional supplementary materials:  crystallographic information; 3D view; checkCIF report

## Figures and Tables

**Table 1:** Hydrogen-bond geometry (Å, °)

*D*—H⋯*A*	*D*—H	H⋯*A*	*D*⋯*A*	*D*—H⋯*A*
O6—H6⋯O2^i^	0.84	2.01	2.854 (2)	178

Symmetry code: (i) 
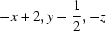
.

**Fig. 1.**
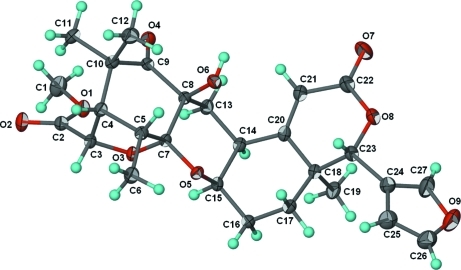
Thermal ellipsoid plot (Barbour, 2001) of C27H32O9 at the 70% probability level. Hydrogen atoms are drawn as spheres of arbitrary radius.

## supplementary crystallographic information

### Experimental

*Lansium domesticum *Corr. (Meliaceae) was collected in Cililin, Bandung, Indonesia, in 2006. The plant was identified by the staff at Department of Biology, Padjadjaran University. The dried and milled seeds of *L.domesticum* (2 kg) was extracted exhaustively by methanol at room temperature. The methanol extract (84 g) was partitioned between *n*-hexane and 10% aqueous methanol to give an *n*-hexane soluble fraction (4 g). The *n*-hexane extract were subjected to column chromatography on silica gel 60 by using step gradient of *n*-hexane and dichloromethane (8:2). The fraction eluted by *n-*hexane/dichloromethane (6:4) was further separated by column chromatography on silica gel (n-hexane:ethyl acetate 7:3) to give kokosanolide (48 mg).

### Refinement

Carbon-bound H-atoms were placed in calculated positions (C–H 0.95–0.98 Å) and were included in the refinement in the riding model approximation, with *U*(H) set to 1.2–1.5*U*(C). The hydroxy H-atom was similarly treated.

### Crystal data

**Table:**

C_27_H_32_O_9_	*F*(000) = 532
*M**_r_* = 500.53	*D*_x_ = 1.399 Mg m^−^^3^
Monoclinic, *P*2_1_	Mo *K*α radiation, λ = 0.71073 Å
Hall symbol: P 2yb	Cell parameters from 8527 reflections
*a* = 8.8326 (1) Å	θ = 2.5–28.3°
*b* = 12.8977 (2) Å	µ = 0.11 mm^−^^1^
*c* = 11.1555 (1) Å	*T* = 123 K
β = 110.777 (1)°	Block, colorless
*V* = 1188.19 (3) Å^3^	0.40 × 0.30 × 0.10 mm
*Z* = 2	

### Data collection

**Table:**

Bruker SMART APEX diffractometer	2760 reflections with *I* > 2σ(*I*)
Radiation source: fine-focus sealed tube	*R*_int_ = 0.020
graphite	θ_max_ = 27.5°, θ_min_ = 2.0°
ω scans	*h* = −11→11
11333 measured reflections	*k* = −16→16
2856 independent reflections	*l* = −14→14

### Refinement

**Table:**

Refinement on *F*^2^	Primary atom site location: structure-invariant direct methods
Least-squares matrix: full	Secondary atom site location: difference Fourier map
*R*[*F*^2^ > 2σ(*F*^2^)] = 0.030	Hydrogen site location: inferred from neighbouring sites
*wR*(*F*^2^) = 0.083	H-atom parameters constrained
*S* = 1.03	*w* = 1/[σ^2^(*F*_o_^2^) + (0.0619*P*)^2^ + 0.1566*P*] where *P* = (*F*_o_^2^ + 2*F*_c_^2^)/3
2856 reflections	(Δ/σ)_max_ = 0.001
331 parameters	Δρ_max_ = 0.32 e Å^−^^3^
1 restraint	Δρ_min_ = −0.21 e Å^−^^3^

### Fractional atomic coordinates and isotropic or equivalent isotropic displacement parameters (Å^2^)

**Table:**

	*x*	*y*	*z*	*U*_iso_*/*U*_eq_	
O1	0.84140 (14)	0.50022 (10)	0.13328 (11)	0.0178 (2)	
O2	0.76288 (15)	0.65296 (11)	0.03392 (13)	0.0216 (3)	
O3	1.14818 (14)	0.54506 (9)	0.20719 (11)	0.0149 (2)	
O4	0.96159 (15)	0.32343 (10)	0.00956 (13)	0.0206 (3)	
O5	1.42245 (14)	0.50619 (9)	0.27176 (11)	0.0153 (2)	
O6	1.34114 (15)	0.35288 (10)	0.07332 (12)	0.0170 (2)	
H6	1.3135	0.2934	0.0428	0.026*	
O7	1.59091 (18)	0.01203 (12)	0.27280 (16)	0.0320 (3)	
O8	1.77358 (16)	0.12432 (10)	0.38724 (14)	0.0236 (3)	
O9	2.20986 (18)	0.18614 (14)	0.66355 (16)	0.0361 (4)	
C1	0.6733 (2)	0.47246 (17)	0.10624 (19)	0.0243 (4)	
H1A	0.6663	0.3989	0.1258	0.036*	
H1B	0.6109	0.4850	0.0154	0.036*	
H1C	0.6293	0.5147	0.1592	0.036*	
C2	0.8676 (2)	0.59050 (14)	0.08735 (16)	0.0161 (3)	
C3	1.04435 (18)	0.60764 (13)	0.10460 (15)	0.0146 (3)	
H3	1.0710	0.6821	0.1276	0.017*	
C4	1.09301 (19)	0.58216 (13)	−0.01273 (15)	0.0147 (3)	
H4	1.0674	0.6416	−0.0743	0.018*	
C5	1.27793 (19)	0.57214 (13)	0.05798 (16)	0.0151 (3)	
H5	1.3274	0.5339	0.0030	0.018*	
C6	1.3650 (2)	0.67574 (14)	0.10056 (18)	0.0199 (3)	
H6A	1.4797	0.6630	0.1498	0.030*	
H6B	1.3158	0.7128	0.1543	0.030*	
H6C	1.3551	0.7177	0.0249	0.030*	
C7	1.27580 (18)	0.50317 (13)	0.16845 (15)	0.0136 (3)	
C8	1.22596 (19)	0.39101 (13)	0.12532 (16)	0.0137 (3)	
C9	1.0573 (2)	0.39205 (13)	0.01658 (16)	0.0146 (3)	
C10	1.0219 (2)	0.47930 (13)	−0.08308 (15)	0.0159 (3)	
C11	0.8397 (2)	0.48559 (15)	−0.16080 (16)	0.0199 (3)	
H11A	0.8027	0.4195	−0.2048	0.030*	
H11B	0.8193	0.5414	−0.2242	0.030*	
H11C	0.7807	0.4999	−0.1029	0.030*	
C12	1.1023 (2)	0.45050 (16)	−0.18142 (17)	0.0220 (4)	
H12A	1.0540	0.3864	−0.2257	0.033*	
H12B	1.2187	0.4403	−0.1367	0.033*	
H12C	1.0849	0.5066	−0.2441	0.033*	
C13	1.2208 (2)	0.32682 (13)	0.23880 (16)	0.0161 (3)	
H13A	1.2015	0.2532	0.2127	0.019*	
H13B	1.1293	0.3506	0.2634	0.019*	
C14	1.3790 (2)	0.33558 (13)	0.35547 (16)	0.0162 (3)	
H14	1.3509	0.3102	0.4297	0.019*	
C15	1.4203 (2)	0.45085 (13)	0.38358 (15)	0.0159 (3)	
H15	1.3358	0.4828	0.4123	0.019*	
C16	1.5849 (2)	0.46840 (14)	0.48766 (16)	0.0186 (3)	
H16A	1.5790	0.4487	0.5717	0.022*	
H16B	1.6121	0.5431	0.4911	0.022*	
C17	1.7188 (2)	0.40598 (13)	0.46466 (17)	0.0171 (3)	
H17A	1.8226	0.4193	0.5355	0.021*	
H17B	1.7306	0.4296	0.3840	0.021*	
C18	1.6839 (2)	0.28830 (13)	0.45591 (16)	0.0159 (3)	
C19	1.6878 (2)	0.24514 (15)	0.58587 (17)	0.0204 (3)	
H19A	1.6774	0.1695	0.5807	0.031*	
H19B	1.5979	0.2746	0.6067	0.031*	
H19C	1.7906	0.2639	0.6529	0.031*	
C20	1.5183 (2)	0.26731 (13)	0.35417 (16)	0.0156 (3)	
C21	1.4975 (2)	0.18237 (14)	0.28188 (17)	0.0197 (3)	
H21	1.3999	0.1746	0.2103	0.024*	
C22	1.6205 (2)	0.10045 (16)	0.30943 (19)	0.0225 (4)	
C23	1.8114 (2)	0.23374 (14)	0.41244 (18)	0.0186 (3)	
H23	1.8107	0.2672	0.3314	0.022*	
C24	1.9817 (2)	0.23557 (16)	0.50764 (19)	0.0212 (4)	
C25	2.0961 (2)	0.31887 (18)	0.53514 (19)	0.0252 (4)	
H25	2.0804	0.3850	0.4950	0.030*	
C26	2.2305 (2)	0.28487 (19)	0.6295 (2)	0.0290 (4)	
H26	2.3264	0.3245	0.6669	0.035*	
C27	2.0560 (3)	0.15765 (18)	0.5880 (2)	0.0329 (5)	
H27	2.0083	0.0923	0.5917	0.040*	

### Atomic displacement parameters (Å^2^)

**Table:**

	*U*^11^	*U*^22^	*U*^33^	*U*^12^	*U*^13^	*U*^23^
O1	0.0136 (5)	0.0176 (6)	0.0218 (6)	0.0018 (5)	0.0058 (5)	0.0033 (5)
O2	0.0158 (6)	0.0207 (6)	0.0260 (6)	0.0053 (5)	0.0045 (5)	0.0052 (5)
O3	0.0122 (5)	0.0162 (6)	0.0147 (5)	0.0043 (4)	0.0027 (4)	0.0011 (4)
O4	0.0149 (6)	0.0150 (6)	0.0271 (6)	−0.0020 (5)	0.0017 (5)	−0.0014 (5)
O5	0.0121 (5)	0.0144 (5)	0.0159 (5)	−0.0007 (4)	0.0006 (4)	−0.0001 (4)
O6	0.0146 (5)	0.0147 (5)	0.0221 (6)	0.0007 (5)	0.0069 (5)	−0.0035 (5)
O7	0.0263 (7)	0.0196 (7)	0.0467 (9)	0.0014 (6)	0.0086 (6)	−0.0097 (6)
O8	0.0197 (6)	0.0176 (6)	0.0296 (7)	0.0049 (5)	0.0043 (5)	−0.0040 (5)
O9	0.0207 (7)	0.0400 (9)	0.0372 (8)	0.0037 (7)	−0.0027 (6)	0.0026 (7)
C1	0.0160 (8)	0.0291 (10)	0.0283 (9)	−0.0012 (7)	0.0084 (7)	0.0054 (8)
C2	0.0154 (7)	0.0169 (8)	0.0152 (7)	0.0018 (6)	0.0045 (6)	−0.0007 (6)
C3	0.0132 (7)	0.0139 (7)	0.0149 (7)	0.0022 (6)	0.0029 (6)	0.0016 (6)
C4	0.0129 (7)	0.0150 (7)	0.0151 (7)	−0.0001 (6)	0.0034 (6)	0.0011 (6)
C5	0.0123 (7)	0.0147 (7)	0.0169 (7)	−0.0010 (6)	0.0035 (6)	−0.0005 (6)
C6	0.0170 (8)	0.0151 (8)	0.0243 (8)	−0.0015 (6)	0.0033 (6)	0.0028 (6)
C7	0.0101 (7)	0.0139 (7)	0.0149 (7)	0.0008 (6)	0.0021 (6)	−0.0012 (6)
C8	0.0104 (7)	0.0126 (7)	0.0165 (7)	−0.0005 (6)	0.0027 (6)	−0.0014 (6)
C9	0.0130 (7)	0.0141 (7)	0.0158 (7)	0.0017 (6)	0.0041 (6)	−0.0031 (6)
C10	0.0146 (7)	0.0174 (8)	0.0138 (7)	0.0004 (6)	0.0027 (6)	−0.0015 (6)
C11	0.0150 (8)	0.0229 (8)	0.0171 (7)	0.0009 (7)	0.0001 (6)	−0.0012 (7)
C12	0.0219 (8)	0.0274 (9)	0.0169 (7)	0.0014 (7)	0.0072 (7)	−0.0029 (7)
C13	0.0131 (7)	0.0140 (7)	0.0189 (8)	−0.0006 (6)	0.0029 (6)	0.0022 (6)
C14	0.0146 (7)	0.0155 (7)	0.0159 (7)	0.0014 (6)	0.0023 (6)	0.0011 (6)
C15	0.0158 (7)	0.0154 (7)	0.0140 (7)	0.0042 (6)	0.0021 (6)	0.0002 (6)
C16	0.0185 (8)	0.0156 (8)	0.0168 (7)	0.0032 (6)	0.0001 (6)	−0.0027 (6)
C17	0.0153 (7)	0.0158 (8)	0.0169 (7)	0.0008 (6)	0.0016 (6)	−0.0001 (6)
C18	0.0137 (7)	0.0160 (8)	0.0163 (7)	0.0023 (6)	0.0033 (6)	0.0001 (6)
C19	0.0206 (8)	0.0220 (9)	0.0160 (8)	0.0033 (7)	0.0034 (6)	0.0046 (7)
C20	0.0140 (7)	0.0157 (7)	0.0162 (7)	0.0017 (6)	0.0043 (6)	0.0029 (6)
C21	0.0163 (8)	0.0187 (8)	0.0213 (8)	0.0025 (7)	0.0032 (6)	−0.0001 (7)
C22	0.0203 (8)	0.0199 (8)	0.0267 (9)	0.0034 (7)	0.0076 (7)	−0.0028 (7)
C23	0.0159 (8)	0.0173 (8)	0.0214 (8)	0.0026 (6)	0.0051 (7)	−0.0020 (7)
C24	0.0169 (8)	0.0244 (9)	0.0217 (8)	0.0025 (7)	0.0059 (7)	−0.0038 (7)
C25	0.0183 (8)	0.0317 (10)	0.0274 (9)	−0.0014 (8)	0.0104 (7)	−0.0025 (8)
C26	0.0176 (9)	0.0388 (12)	0.0300 (10)	−0.0009 (8)	0.0075 (8)	−0.0078 (9)
C27	0.0204 (9)	0.0289 (10)	0.0392 (11)	0.0034 (8)	−0.0022 (8)	0.0025 (9)

### Geometric parameters (Å, °)

**Table:**

O1—C2	1.325 (2)	C11—H11B	0.9800
O1—C1	1.451 (2)	C11—H11C	0.9800
O2—C2	1.212 (2)	C12—H12A	0.9800
O3—C3	1.4344 (19)	C12—H12B	0.9800
O3—C7	1.4473 (19)	C12—H12C	0.9800
O4—C9	1.207 (2)	C13—C14	1.539 (2)
O5—C7	1.3959 (18)	C13—H13A	0.9900
O5—C15	1.443 (2)	C13—H13B	0.9900
O6—C8	1.4255 (19)	C14—C20	1.517 (2)
O6—H6	0.8400	C14—C15	1.536 (2)
O7—C22	1.208 (3)	C14—H14	1.0000
O8—C22	1.358 (2)	C15—C16	1.521 (2)
O8—C23	1.455 (2)	C15—H15	1.0000
O9—C26	1.360 (3)	C16—C17	1.526 (2)
O9—C27	1.370 (2)	C16—H16A	0.9900
C1—H1A	0.9800	C16—H16B	0.9900
C1—H1B	0.9800	C17—C18	1.545 (2)
C1—H1C	0.9800	C17—H17A	0.9900
C2—C3	1.520 (2)	C17—H17B	0.9900
C3—C4	1.551 (2)	C18—C20	1.524 (2)
C3—H3	1.0000	C18—C23	1.545 (2)
C4—C5	1.547 (2)	C18—C19	1.542 (2)
C4—C10	1.556 (2)	C19—H19A	0.9800
C4—H4	1.0000	C19—H19B	0.9800
C5—C7	1.525 (2)	C19—H19C	0.9800
C5—C6	1.531 (2)	C20—C21	1.334 (2)
C5—H5	1.0000	C21—C22	1.468 (2)
C6—H6A	0.9800	C21—H21	0.9500
C6—H6B	0.9800	C23—C24	1.503 (2)
C6—H6C	0.9800	C23—H23	1.0000
C7—C8	1.539 (2)	C24—C27	1.352 (3)
C8—C13	1.527 (2)	C24—C25	1.431 (3)
C8—C9	1.552 (2)	C25—C26	1.350 (3)
C9—C10	1.534 (2)	C25—H25	0.9500
C10—C11	1.536 (2)	C26—H26	0.9500
C10—C12	1.548 (2)	C27—H27	0.9500
C11—H11A	0.9800		
			
C2—O1—C1	116.25 (14)	H12B—C12—H12C	109.5
C3—O3—C7	108.49 (12)	C8—C13—C14	112.08 (13)
C7—O5—C15	114.02 (12)	C8—C13—H13A	109.2
C8—O6—H6	109.5	C14—C13—H13A	109.2
C22—O8—C23	116.86 (14)	C8—C13—H13B	109.2
C26—O9—C27	106.34 (17)	C14—C13—H13B	109.2
O1—C1—H1A	109.5	H13A—C13—H13B	107.9
O1—C1—H1B	109.5	C20—C14—C15	115.40 (14)
H1A—C1—H1B	109.5	C20—C14—C13	116.45 (14)
O1—C1—H1C	109.5	C15—C14—C13	108.80 (13)
H1A—C1—H1C	109.5	C20—C14—H14	105.0
H1B—C1—H1C	109.5	C15—C14—H14	105.0
O2—C2—O1	124.55 (16)	C13—C14—H14	105.0
O2—C2—C3	122.41 (16)	O5—C15—C16	106.52 (14)
O1—C2—C3	113.02 (14)	O5—C15—C14	112.16 (13)
O3—C3—C2	110.89 (13)	C16—C15—C14	113.15 (13)
O3—C3—C4	105.31 (12)	O5—C15—H15	108.3
C2—C3—C4	116.24 (13)	C16—C15—H15	108.3
O3—C3—H3	108.0	C14—C15—H15	108.3
C2—C3—H3	108.0	C15—C16—C17	112.39 (14)
C4—C3—H3	108.0	C15—C16—H16A	109.1
C5—C4—C3	98.39 (12)	C17—C16—H16A	109.1
C5—C4—C10	110.50 (13)	C15—C16—H16B	109.1
C3—C4—C10	115.04 (14)	C17—C16—H16B	109.1
C5—C4—H4	110.8	H16A—C16—H16B	107.9
C3—C4—H4	110.8	C16—C17—C18	112.30 (15)
C10—C4—H4	110.8	C16—C17—H17A	109.1
C7—C5—C6	114.03 (14)	C18—C17—H17A	109.1
C7—C5—C4	98.47 (13)	C16—C17—H17B	109.1
C6—C5—C4	114.11 (14)	C18—C17—H17B	109.1
C7—C5—H5	109.9	H17A—C17—H17B	107.9
C6—C5—H5	109.9	C20—C18—C23	107.41 (13)
C4—C5—H5	109.9	C20—C18—C19	109.36 (14)
C5—C6—H6A	109.5	C23—C18—C19	110.80 (14)
C5—C6—H6B	109.5	C20—C18—C17	109.81 (14)
H6A—C6—H6B	109.5	C23—C18—C17	108.41 (14)
C5—C6—H6C	109.5	C19—C18—C17	110.97 (15)
H6A—C6—H6C	109.5	C18—C19—H19A	109.5
H6B—C6—H6C	109.5	C18—C19—H19B	109.5
O5—C7—O3	109.63 (12)	H19A—C19—H19B	109.5
O5—C7—C5	112.14 (13)	C18—C19—H19C	109.5
O3—C7—C5	104.93 (13)	H19A—C19—H19C	109.5
O5—C7—C8	111.30 (13)	H19B—C19—H19C	109.5
O3—C7—C8	106.12 (12)	C21—C20—C14	122.97 (16)
C5—C7—C8	112.32 (13)	C21—C20—C18	119.08 (15)
O6—C8—C13	112.70 (13)	C14—C20—C18	117.20 (14)
O6—C8—C7	106.58 (12)	C20—C21—C22	122.12 (16)
C13—C8—C7	109.87 (13)	C20—C21—H21	118.9
O6—C8—C9	108.09 (13)	C22—C21—H21	118.9
C13—C8—C9	110.49 (13)	O7—C22—O8	118.50 (17)
C7—C8—C9	108.97 (13)	O7—C22—C21	123.59 (18)
O4—C9—C10	122.19 (15)	O8—C22—C21	117.77 (17)
O4—C9—C8	120.02 (15)	O8—C23—C24	104.80 (14)
C10—C9—C8	117.72 (14)	O8—C23—C18	111.08 (14)
C9—C10—C11	110.56 (14)	C24—C23—C18	115.43 (15)
C9—C10—C12	108.10 (14)	O8—C23—H23	108.4
C11—C10—C12	105.35 (13)	C24—C23—H23	108.4
C9—C10—C4	108.54 (13)	C18—C23—H23	108.4
C11—C10—C4	113.22 (14)	C27—C24—C25	106.02 (18)
C12—C10—C4	110.95 (14)	C27—C24—C23	125.89 (18)
C10—C11—H11A	109.5	C25—C24—C23	128.07 (18)
C10—C11—H11B	109.5	C26—C25—C24	106.4 (2)
H11A—C11—H11B	109.5	C26—C25—H25	126.8
C10—C11—H11C	109.5	C24—C25—H25	126.8
H11A—C11—H11C	109.5	C25—C26—O9	110.73 (19)
H11B—C11—H11C	109.5	C25—C26—H26	124.6
C10—C12—H12A	109.5	O9—C26—H26	124.6
C10—C12—H12B	109.5	C24—C27—O9	110.5 (2)
H12A—C12—H12B	109.5	C24—C27—H27	124.7
C10—C12—H12C	109.5	O9—C27—H27	124.7
H12A—C12—H12C	109.5		
			
C1—O1—C2—O2	5.7 (2)	C3—C4—C10—C12	−169.29 (14)
C1—O1—C2—C3	−172.55 (15)	O6—C8—C13—C14	66.52 (17)
C7—O3—C3—C2	138.99 (13)	C7—C8—C13—C14	−52.18 (17)
C7—O3—C3—C4	12.48 (16)	C9—C8—C13—C14	−172.45 (13)
O2—C2—C3—O3	158.88 (15)	C8—C13—C14—C20	−81.44 (18)
O1—C2—C3—O3	−22.83 (19)	C8—C13—C14—C15	51.05 (18)
O2—C2—C3—C4	−80.9 (2)	C7—O5—C15—C16	−176.46 (13)
O1—C2—C3—C4	97.37 (17)	C7—O5—C15—C14	59.23 (17)
O3—C3—C4—C5	−37.89 (15)	C20—C14—C15—O5	80.22 (17)
C2—C3—C4—C5	−161.05 (14)	C13—C14—C15—O5	−52.82 (17)
O3—C3—C4—C10	79.47 (16)	C20—C14—C15—C16	−40.3 (2)
C2—C3—C4—C10	−43.69 (19)	C13—C14—C15—C16	−173.35 (13)
C3—C4—C5—C7	47.08 (14)	O5—C15—C16—C17	−74.21 (17)
C10—C4—C5—C7	−73.71 (15)	C14—C15—C16—C17	49.5 (2)
C3—C4—C5—C6	−74.10 (17)	C15—C16—C17—C18	−57.93 (19)
C10—C4—C5—C6	165.11 (15)	C16—C17—C18—C20	54.44 (19)
C15—O5—C7—O3	57.64 (17)	C16—C17—C18—C23	171.52 (13)
C15—O5—C7—C5	173.77 (13)	C16—C17—C18—C19	−66.58 (18)
C15—O5—C7—C8	−59.45 (16)	C15—C14—C20—C21	−150.03 (16)
C3—O3—C7—O5	139.56 (13)	C13—C14—C20—C21	−20.6 (2)
C3—O3—C7—C5	18.95 (16)	C15—C14—C20—C18	40.0 (2)
C3—O3—C7—C8	−100.14 (14)	C13—C14—C20—C18	169.39 (14)
C6—C5—C7—O5	−39.71 (19)	C23—C18—C20—C21	25.9 (2)
C4—C5—C7—O5	−160.94 (13)	C19—C18—C20—C21	−94.40 (19)
C6—C5—C7—O3	79.23 (16)	C17—C18—C20—C21	143.61 (17)
C4—C5—C7—O3	−42.00 (15)	C23—C18—C20—C14	−163.70 (15)
C6—C5—C7—C8	−165.93 (13)	C19—C18—C20—C14	75.99 (18)
C4—C5—C7—C8	72.83 (15)	C17—C18—C20—C14	−46.00 (19)
O5—C7—C8—O6	−67.25 (16)	C14—C20—C21—C22	−160.32 (17)
O3—C7—C8—O6	173.53 (12)	C18—C20—C21—C22	9.5 (3)
C5—C7—C8—O6	59.42 (16)	C23—O8—C22—O7	170.30 (18)
O5—C7—C8—C13	55.14 (17)	C23—O8—C22—C21	−13.9 (2)
O3—C7—C8—C13	−64.08 (15)	C20—C21—C22—O7	157.6 (2)
C5—C7—C8—C13	−178.18 (13)	C20—C21—C22—O8	−18.0 (3)
O5—C7—C8—C9	176.32 (12)	C22—O8—C23—C24	175.76 (15)
O3—C7—C8—C9	57.11 (15)	C22—O8—C23—C18	50.5 (2)
C5—C7—C8—C9	−57.00 (17)	C20—C18—C23—O8	−54.44 (18)
O6—C8—C9—O4	100.48 (17)	C19—C18—C23—O8	64.95 (18)
C13—C8—C9—O4	−23.3 (2)	C17—C18—C23—O8	−173.05 (14)
C7—C8—C9—O4	−144.06 (15)	C20—C18—C23—C24	−173.55 (15)
O6—C8—C9—C10	−76.61 (17)	C19—C18—C23—C24	−54.2 (2)
C13—C8—C9—C10	159.65 (14)	C17—C18—C23—C24	67.85 (19)
C7—C8—C9—C10	38.84 (18)	O8—C23—C24—C27	−23.5 (3)
O4—C9—C10—C11	17.8 (2)	C18—C23—C24—C27	99.1 (2)
C8—C9—C10—C11	−165.21 (13)	O8—C23—C24—C25	158.54 (18)
O4—C9—C10—C12	−97.08 (19)	C18—C23—C24—C25	−78.9 (2)
C8—C9—C10—C12	79.95 (17)	C27—C24—C25—C26	0.2 (2)
O4—C9—C10—C4	142.50 (16)	C23—C24—C25—C26	178.52 (18)
C8—C9—C10—C4	−40.47 (19)	C24—C25—C26—O9	0.1 (2)
C5—C4—C10—C9	59.63 (17)	C27—O9—C26—C25	−0.4 (2)
C3—C4—C10—C9	−50.65 (17)	C25—C24—C27—O9	−0.4 (2)
C5—C4—C10—C11	−177.22 (14)	C23—C24—C27—O9	−178.81 (18)
C3—C4—C10—C11	72.50 (17)	C26—O9—C27—C24	0.5 (2)
C5—C4—C10—C12	−59.01 (17)		

### Hydrogen-bond geometry (Å, °)

**Table:**

*D*—H···*A*	*D*—H	H···*A*	*D*···*A*	*D*—H···*A*
O6—H6···O2^i^	0.84	2.01	2.854 (2)	178

Symmetry codes: (i) −*x*+2, *y*−1/2, −*z*.
